# Physiotherapy practice in women’s health: awareness and attitudes of obstetricians and gynecologists in Ghana

**DOI:** 10.1186/s12905-023-02705-5

**Published:** 2023-12-11

**Authors:** Yaa Boatemaa Koranteng, Kwame Adu-Bonsaffoh, Bertha Oppong-Yeboah

**Affiliations:** 1 Department of Physiotherapy, Korle-Bu Teaching Hospital, Accra, Ghana; 2 Department of Obstetrics and Gynecology, University of Ghana Medical School, Accra, Ghana; 3 Department of Physiotherapy, University of Health and Allied Sciences, Ho, Ghana

**Keywords:** Physiotherapy, Women’s health, Awareness, Attitude, Obstetricians, Gynecologists

## Abstract

**Background:**

Physiotherapy is relatively well integrated into women’s health in many high-income countries (HICs) as compared to low- and middle- countries (LMICs) like Ghana. Suboptimal integration of physiotherapy in modern obstetrics and gynecology especially in low resource settings is partly due to issues related to the awareness and attitudes of referring physicians. This study assessed the awareness and attitude levels towards physiotherapy in women’s health among obstetricians/gynecologists and factors associated with its utilization in Ghana.

**Methods:**

A cross-sectional study was conducted among obstetricians/gynecologists working at a tertiary hospital in Ghana using an “Awareness and Attitude Questionnaire” adapted from a standardized questionnaire. Chi-square test or Fisher exact test was performed and logistic regression was used to assess the association between doctors’ awareness level of physiotherapy’s role in women’s health and years of clinical practice.

**Results:**

Sixty-one (61) respondents comprising 7 consultants, 20 senior residents and 34 junior residents, with age median age of 35 years (range: 29–65 years) were recruited. There were more males than females (82% versus 18%) with a mean (SD) duration of practice of (9.41 ± 4.71) years. The participants reported a considerable awareness of physiotherapists’ role in obstetrics (between 72.1% for intrapartum to 91.8% for postnatal) but wide variation in gynecology (from 19.7% in PID to 95. 1% in uterine prolapse). Consultants were more (71.4%) aware of the role of physiotherapy in antenatal care and gynecology while senior residents had more awareness in intrapartum and postnatal care. Junior residents generally showed lowest awareness levels. Duration of clinical practice (≥ 10years) was not significantly associated with doctors’ awareness regarding the importance of physiotherapy in childbirth. There were mixed findings concerning doctors’ attitudes toward physiotherapy: (1) 41% indicated that physiotherapists have been effective in their inter-professional relationship; (2) none of the doctors strongly agreed that physiotherapy may not contribute significantly to the complete well-being of gynecological patients. The main factors influencing utilization of physiotherapy were the perceived notion of non-availability of physiotherapists to cover various wards and physiotherapists not attending ward rounds with doctors to facilitate more education on the scope of physiotherapy practice.

**Conclusion:**

Although obstetricians/gynecologists showed appreciable awareness and attitudes towards physiotherapy, there remains a considerable gap in provider education to ensure optimal utilization of physiotherapy in contemporary obstetrics and gynecology. Further research is recommended to assess implementation challenges associated with regular utilization of physiotherapy services in women’s health in the hospital.

## Background

Physiotherapy plays a significant adjunctive role at all stages of healthcare in various medical specialties including women’s health [1]. It is not a substitute but a complimentary intervention to other forms of clinical management to enhance efficiency and quality of medical care. Physiotherapists as part of the health care team play an important role in reducing hospital stay duration, recovery period and rehabilitation for a better quality of life [2]. There is a common misconception that physiotherapy’s importance is limited to musculoskeletal conditions but the scope is wider and incorporates other specialty areas such as women’s health care [3]. Physiotherapy in women’s health is pivotal in treating a wide variety of obstetric and gynecological issues [4–7]. For instance, pregnancy is characterized by disturbing physiological changes (physical and emotional) and childbirth further compounds the stress. However, various physiotherapy interventions including breathing exercises and relaxation may be both preventive and therapeutic in women’s health especially during labour [8].

Physiotherapists are efficient in managing some complications of pregnancy and childbirth such as pelvic floor dysfunction and low back pain via manual therapy, exercise and or electrotherapeutic modalities [6, 9]. Though physiotherapy is vital in maternal health, it is still not widely practiced in low and middle income countries and remains underutilized [10–13]. Utilization of individual professional skills in the multidisciplinary approach depends on co-operation between healthcare team members and the extent to which they value the knowledge of one another [4].

Previous studies have recorded low referral rate and poor utilization of physiotherapy by obstetricians and gynecologists [11, 12, 14] and suboptimal knowledge concerning the preventive role of physiotherapy during antenatal and postnatal care has been implicated [12]. Physicians, including obstetricians and gynecologists are at the ‘top of the pyramid’ of health professionals, and have profound influences on other health workers including physiotherapists, in terms of making the appropriate referrals [15]. The issue of delayed involvement of physiotherapy by most physicians has been of great concern to physiotherapists [16, 17]. In order for patients to be referred to other members of the multidisciplinary team, health professionals need to understand each other’s role and contribution towards patients’ care [18].

Though sufficient evidence exist in international literature concerning the role and effectiveness of physiotherapy in the practice of obstetrics and gynecology [19, 20], considerable awareness of its scope remains limited in Ghana. More recently, an important initiative to actively integrate physiotherapy in urogynecology commenced at the largest tertiary hospital in Ghana and two young physiotherapists received formal training in pelvic floor rehabilitation [21]. Optimal integration of physiotherapy services in maternal health by obstetricians/gynecologists depends on their knowledge on the specific conditions amenable to physiotherapy. In Ghana, there is limited research on the role of physiotherapy in women’s health. However, there is evidence that obstetric events contribute significantly to the burden of urinary incontinence and pelvic organ prolapse which require adjunctive physiotherapy. In addition, the obstetrician’s knowledge and awareness of the role of physiotherapy in Women’s health has not been reported. In a descriptive study of seven hospitals in Nigeria, Odunaiya et al. determined that the obstetricians and gynecologists demonstrated limited knowledge about specific conditions amenable to physiotherapy treatment although they had general knowledge concerning the role of physiotherapy in women’s health [4].

The aim of this study was to evaluate the level of awareness and attitudes of obstetricians/gynecologists towards physiotherapy in women’s health and the factors influencing its utilization in Ghana. This study highlights significant clinical insights into the management of women’s health issues during the antenatal, intrapartum and postpartum periods and gynecological care in Ghana.

## Methods

### Study design and site

The study was a cross sectional study, conducted at the Department of Obstetrics and Gynecology at the Korle Bu Teaching Hospital (KBTH), in Accra, Ghana. KBTH is the largest tertiary Hospital in the southern part of Ghana, and an accredited training facility for both the West African and Ghana College of Physicians and Surgeons. It is currently the leading national referral center in Ghana comprising various medical specialty departments. The Department of Obstetrics and Gynecology of the hospital is divided into five units (Teams A, B, C, D and E) and has a bed capacity of 372 (97 and 275 beds for Gynecology and Obstetrics respectively [22]. A senior consultant heads each unit with other consultants and doctors (comprising senior residents, junior residents and house officers) equally distributed among the various units. The consultants are usually permanent while the other doctors (mainly residents) rotate through the teams. Each unit has its specific clinic, theatre and grand ward round days.

### Study participants

The participants for the study were medical doctors (consultants, senior residents and junior residents) in obstetrics and gynecology who were working at the hospital. A consultant obstetrician/gynecologist is a medical doctor of the highest rank who deals with women’s health problems relating to the female reproductive system. Senior and junior residents are medical doctors in residency training to become consultants and specialists respectively. At the time of the study, there were 17 consultants, 23 senior residents and about 55 junior residents in the Department of Obstetrics and Gynecology, KBTH. The inclusion criteria were obstetricians/gynecologists (consultants) and clinicians who were pursuing their residency training program and had worked at the Department of Obstetrics and Gynecology for at least one year. Specific exclusion criteria were failure to provide informed consent, house officers undertaking their rotations at the Department of Obstetrics and Gynecology and resident doctors who had spent less than 12 months into their residency training. Also, doctors who were on leave were excluded from the study.

### Data collection and variables

Prior to the data collection, a formal protocol presentation was done at the Department of Obstetrics and Gynecology to all the clinical staff including the doctors, nurses and midwives during one of the their clinical meetings. Convenient sampling method was used in recruiting the study participants based on their accessibility, availability at the time of the study and willingness to participate. The KBTH was chosen as the study site because it is the largest residency training center for obstetrics and gynecology in Ghana and manages high number of obstetric and gynecological cases. Based on the nature of the study, the sampling procedure employed all the available and willing doctors (consultants, senior and residents ) working at the KBTH with reference to the inclusion and exclusion criteria.

**Fig. 1:**
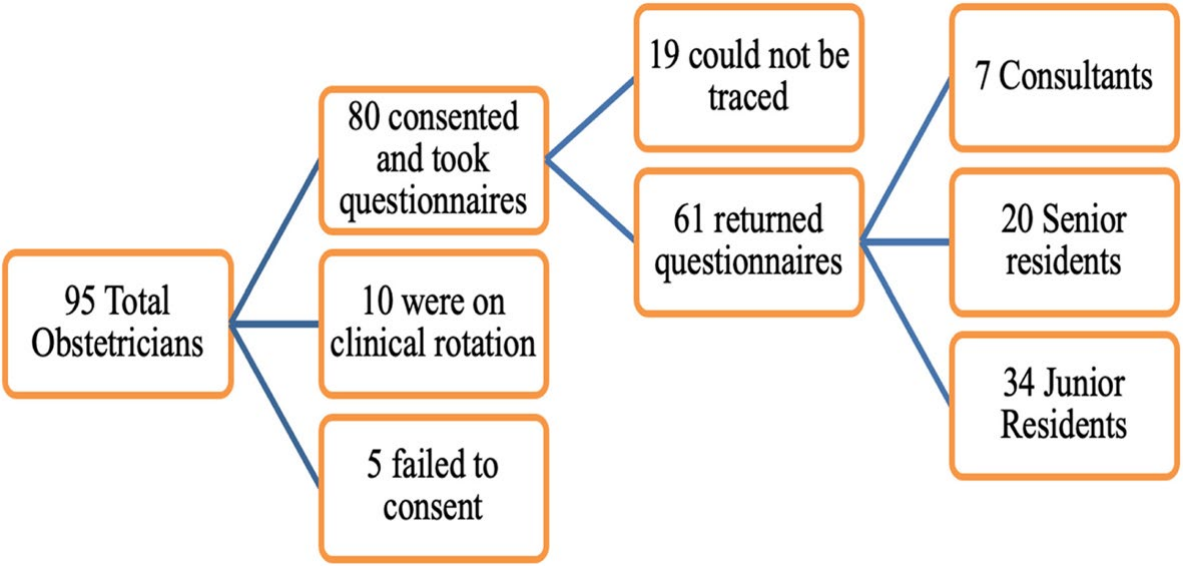
Flow chart showing the study participants included in the study

An “Awareness and Attitude Questionnaire” was adapted from a standardized questionnaire used in a previous work in the subregion [4]. The questionnaire comprised three sections exploring socio-demographic characteristics, awareness of physiotherapy, attitude and factors influencing utilization of physiotherapy among obstetricians and gynecologists. The awareness of physiotherapy in obstetrics and gynecology had responses as: ‘yes’, ‘no’ and ‘not sure’ while the attitude scale was in Likert (‘strongly agree’, ‘agree’, ‘somewhat’, ‘strongly disagree’ and ‘disagree’). The study participants were given the questionnaire to complete during their regular morning meetings held in the conference room at the maternity unit from Monday to Friday. Some of the questionnaire were also distributed at the obstetric clinic, gynecology clinic and on their respective wards. Participation in the study was voluntary and participants were informed that they were free to withdraw from the study at any time. Return visits and contact follow up were used to collect the completed questionnaires from doctors who were not able to complete the questionnaire immediately. Figure 1 indicates the flow chart for recruiting the study participants.

### Data analysis

Data were entered into a Microsoft excel and analyzed using R statistical package (version 3.6.3 R Core Team, Vienna, Austria). Descriptive statistics of frequency and percentages were used to determine the awareness and attitude of the doctors towards physiotherapy in obstetrics and gynecology. Chi- square test or Fisher exact test of association was used to compare the association between awareness and categories of the study participants (i.e. position: consultants, senior and junior residents). Logistic regression was used to determine association between the doctors awareness level and years of practice. We adjusted for doctor’s position, sex and age. Statistical significance was set at *p* < 0.05.

## Results

Over the study period, sixty-one (64.2%) obstetricians/gynecologists (out of a total of 95) including resident doctors participated comprising, 7 (11.5%) consultants, 20 (32.8%) senior residents and 34 (55.7%) junior residents. The median age of the doctors was 35 years (range: 29-65years) and the mean (± SD) duration of practice was (9.41 ± 4.71) years (Range: 4 and 35). Most of the participants were residents [54 (88.5%)] with 7 consultants constituting 11.5%. There were 50 (82.0%) and 11 (18.0%) male and female doctors respectively with most of them [38 (62.3%0] having practiced medicine for 6 to 10 years (Table 1). Among the doctors 55.7% (*n* = 34) and 44.3% (*n* = 27) had practiced for less than 10 and 10 or more years respectively.

**Table 1:** Demographic characteristics of study participants

Variable(s)	Number	Percentage
Position		
Junior Resident	34	55.7
Senior Resident	20	32.8
Consultant	7	11.5
Gender		
Male	50	82.0
Female	11	18.0
Age		
< 30	3	4.9
30–39	41	67.2
40–49	16	26.2
≥ 50	1	1.6
Years of practice		
1–5	7	11.5
6–10	38	62.3
11–15	12	12.7
16–20	3	4.9
> 20	1	1.6

### Awareness of physiotherapy’s role in obstetrics and gynecology

Majority of the doctors showed high awareness of the role of physiotherapy in all categories of obstetric care ranging from 72.1 to 91.8% with postnatal period being the highest.

There were mixed results for awareness concerning the role of physiotherapy in specific or selected gynecological conditions (Table 2). Over 95% (*n* = 58) of the doctors reported the need for physiotherapy in managing uterine prolapse whiles 19.7% (*n* = 12) were aware of the role that physiotherapy plays in the management of pelvic inflammatory disease. There was a moderate awareness of 57.4% (*n* = 35) reported for role of physiotherapy following hysterectomy.

**Table 2:** Doctors’ awareness of physiotherapy’s role in obstetric and gynecological practice

Does physiotherapy has a role in the various categories in obstetrics and gynecology?				
Categories	Yes n (%)	No n (%)	Not sure n (%)	Total n (%)
Obstetric practice				
Antenatal^a^	48(78.7)	2(3.3)	10 (16.4)	60(98.4)
Parturition	44 (72.1)	2 (3.3)	15(24.6)	61(100)
Postnatal	56 (91.8)	1(1.6)	4 (6.6)	61 (100)
Gynecological practice				
Pelvic inflammatory disease^a^	12 (19.7)	19 (31.1)	29(47.5)	60 (98.4)
Uterine prolapse	58 (95.1)	2 (3.3)	1 (1.6)	61(100)
Hysterectomy	35 (57.4)	10 (16.4)	16 (26.2)	61 (100)
Cervical incompetence	15 (24.6)	19 (31.1)	27 (44.3)	61 (100)

^a^1 (1.6%) participant each did not respond to the question on both antenatal and pelvic inflammatory disease

**Table 3:** Doctors’ attitudes to physiotherapy in obstetrics and gynecology

Statement	SA N (%)	A N (%)	SW N (%)	D N (%)	SD N (%)
Regarding physiotherapy					
May not contribute significantly to complete well-being of obstetric patients	1(1.6)	1(1.6)	5(8.2)	15(24.6)	39(63.9)
May not contribute significantly to complete well-being of gynecological patients	0(0.0)	1(1.6)	4(6.6)	15(24.6)	41(67.2)
May not contribute significantly to complete well-being with drugs and instructions	1(1.6)	6(9.8)	4(6.6)	24(39.3)	26(42.6)
Is too expensive to be afforded by my patients	3(4.9)	6(9.8)	18(29.5)	18(29.5)	16(26.2)
Is time-demanding	3(4.9)	24(39.3)	11(18.0)	14(23.0)	9(14.8)
Regarding physiotherapists					
Should be allowed to attend the labour ward	12(19.7)	25(41.0)	15(24.6)	9(14.8)	0(0.0)
Should be allowed to attend some surgical operations gynecological patients	5(8.2)	22(36.1)	16(26.2)	15(24.6)	3(4.9)
Are not competent to manage my patients	1(1.6)	1(1.6)	1 (1.6)	19(31.1)	39(63.9)
Will cause harm to my patients	1(1.6)	0(0.0)	1(1.6)	18(29.5)	41(67.2)
Have been effective in their inter-personal relationship^a^	6(9.8)	19(31.1)	14(23.0	13(21.3)	6(9.8)

*SA *Strongly agree; *A *Agree; *SW *Somewhat; *D *Disagree; *SD *Strongly disagree

^a^3(4.9%) participants did not respond

### Attitude towards physiotherapy in obstetric and gynecology

In general, the doctors thought physiotherapists are proficient in the obstetrics and gynecology rehabilitation team suggestive of positive attitude. However, there were specific areas where attitudes were judged negative. For instance, only 12 participants (19.7%) strongly agreed to physiotherapists’ involvement during childbirth whiles 25 (41.0%) agreed [thus, 60.7% (*n* = 37) agreed in total]. This indicates that approximately 40% do not agree to the need for the involvement of physiotherapists during childbirth. Also, 25 (41%) indicated that physiotherapists have been effective in their inter-professional relationship (Table 3). Most of the obstetricians 56 (91.8%) and 54 (88.5%) alluded to the relevance of physiotherapy in gynecology and obstetrics respectively. None of the doctors strongly agreed that physiotherapy may not contribute significantly to complete well-being of gynecological patients although 1.6% (*n* = 1) agreed. Also, 2 doctors (3.3%) agreed that physiotherapy may not contribute significantly to complete well-being of obstetric patients. Table 3 indicates the full spectrum of doctors’ attitudes towards physiotherapy in obstetrics and gynecology.

#### Factors associated with utilization of physiotherapy

Overall, the doctors reported to have positive influences on their use of physiotherapy. However, the main factors influencing their utilization of physiotherapy was physiotherapists’ non-availability in enough numbers to cover the obstetrics and gynecology wards. According to the study participants, only 6.6% (*n* = 4) of physiotherapists attend ward rounds with doctors (Table 4). Similarly, 6.6% (*n* = 4) indicated that there were enough physiotherapist to cover both obstetrics and gynecology wards. Concerning previous working experience with physiotherapy, 50.8% (*n* = 31) and 45.9% (*n* = 28) had worked with physiotherapists in managing obstetric and gynecological patients respectively. On the other hand, 44.3% (*n* = 27) and 47.5% (*n* = 29) had not previously worked with physiotherapist in managing obstetric and gynecological patients.

**Table 4:** Factors influencing doctors’ utilization of physiotherapy in obstetrics and gynaecology

STATEMENT	YES N (%)	NO N (%)	NOT SURE N (%)
Is physiotherapy is too expensive?	11(18.0)	31(50.8)	19(31.1)
Are there enough physiotherapists to cover the obstetrics and gynecology ward?	4(6.6)	27(44.3)	30(49.2)
Has physiotherapy worsened the condition of your patient before?	0(0.0)	56(91.8)	5(8.2)
Is there physiotherapy degree program in college of training?	48(78.7)	7(11.1)	6(9.8)
Are physiotherapist posted in the hospital?^a^	14(23.0)	24(39.3)	21(34.4)
Do physiotherapists go on ward rounds with doctors?	4(6.6)	55(90.2)	2(3.3)
Have you worked with a physiotherapist in managing obstetric patients?	31(50.8)	27(44.3)	39(4.9)
Have you worked with a physiotherapist in managing gynecological patients?	28(45.9)	29(47.5)	4(6.6)
Is there physiotherapy training or department in your hospital?	54(88.5)	5(8.2)	2(3.3)
Do you have physiotherapist as a close friend	30(49.2)	27(44.3)	4(6.6)
Is there physiotherapy clinic within the vicinity of your hospital of practice?	53(86.9)	3(4.9)	5(8.2)

^a^2(3.3%) of the participants did not respond

### Association between doctors’ category and awareness level of physiotherapy in obstetrics and gynecology

There were important findings relating doctors categories and their awareness of the role physiotherapy in obstetrics and gynecology (Table 5). Generally, consultants had more awareness levels on the role of physiotherapy in antenatal care compared to senior residents and junior residents (85.7% versus 80.0% and 78.8% respectively). On the other hand, senior residents reported higher awareness in parturition or childbirth compared to consultants (85.0% versus 71.4%) and postnatal (100.0% versus 85.7% respectively). In terms of gynecology, consultants generally showed higher awareness compared to senior and junior residents in the management of PID (28.6% versus 25.0 and 15.2% respectively), hysterectomy (85.7% versus 55.0% and 52.9% respectively) and cervical incompetence (28.6% versus 20.0% and 26.5% respectively). However, there was no statistical differences between the consultants and residents concerning the awareness of the physiotherapy’s role in women’s health.

**Table 5:** Awareness of physiotherapy’s role in obstetrics and gynecology by different categories of doctors practicing in women’s health

Clinical areas in women’s health for physiotherapy	Junior residents (*n* = 34)	Senior residents (*n* = 20)	Consultants (*n* = 7)	X^2^	*P* value
Antenatal					
Yes	26 (78.8)	16 (80.0)	6 (85.7)	0.173	0.917
No/not sure	7 (21.2)	4 (20.0)	1 (14.3)		
Parturition					
Yes No/not sure	22 (64.7) 12 (35.3)	17 (85.0) 3 (15.0)	5 (71.4) 2 (28.6)	2.582	0.275
Postnatal					
Yes No/not sure	30 (88.2) 4 (11.8)	20 (100.0) 0	6 (85.7) 1 (14.3)	2.706	0.259
Pelvic inflammatory disease					
Yes No/not sure	5 (15.2) 28 (84.8)	5 (25.0) 15 (75.0)	2 (28.6) 4 (71.4)	1.119	0.572
Uterine prolapse					
Yes No/not sure	31 (91.2) 3 (8.8)	20 (100.0)	7 (100.0)	2.506	0.286
Hysterectomy					
Yes No/not sure	18 (52.9) 16 (47.1)	11 (55.0) 9 (45.0)	6 (85.7) 14.3)	2.6182	0.270
Cervical incompetence					
Yes No/not sure	9 (26.5) 25 (73.5)	4 (20.0) 16 (80.0)	2 (28.6) 5 (71.6)	0.352	0.839

Postnatal period and uterine prolapse were excluded from the logistic regression because the participants reported overwhelming relevance of physiotherapy in their management (91.8% and 95.1% respectively). Years of practice for ten years or more was associated with 3.5 times increased odds of doctor’s awareness concerning the role of physiotherapy during childbirth (OR=3.560, 95%CI: 1.070-14.220) in the unadjusted model (Table 6). However, the significance disappeared in the adjusted model. Similarly, practicing for ten years or more showed increased tendency for high awareness of the role of physiotherapy following hysterectomy, however, this did not reach statistical significance in both the unadjusted and adjusted models.

**Table 6:** Association between years of clinical practice and doctors’ awareness of the role of physiotherapy in women’s health

Clinical areas in women’s health	Awareness of physiotherapy’ role	unadjusted OR 95%CI	*P* value	unadjusted OR 95%CI	*P* value
**Antenatal**					
< 10 years working experience	27 (81.8)	Ref.	0.697	0.24 (0.2, 2.47)	0.248
≥ 10 years working experience	21 (77.8)	0.78 (0.21, 2.82)			
**Parturition**					
** <** 10 years working experience	21 (61.8)	Ref.	0.049	2.85 (0.27, 40.03)	0.396
≥ 10 years working experience	23 (85.2)	3.56 (1.07, 14.22)			
**PID**					
< 10 years working experience	5 (15.2)	Ref.	0.247	0.92 (0.08, 9.66)	0.945
≥ 10 years working experience	7 (84.8)	2.14 (0.60, 8.18)			
**Hysterectomy**					
< 10 years working experience	16 (47.1)	Ref.	0.070	3.63 (0.50, 33.90)	0.215
≥ 10 years working experience	19 (70.4)	2.67 (0.94, 8.06)			
**Cervical incompetence**					
< 10 years working experience	8 (23.5)	Ref.	0.829	2.3 (0.28, 21.15)	0.697
≥ 10 years working experience	7 (25.9)	1.14 (0.35, 3.69)			

## Discussion

In this hospital-based study, the obstetricians/gynecologists demonstrated high awareness of the role of physiotherapy in obstetrics (between 72.1 and 91.8%) in all the aspects of maternal care with the highest occurrence associated with postnatal care. This is consistent with the 68% of awareness regarding postnatal exercises determined by Munawar et al. in Pakistan [23]. For specific gynecological conditions, mixed findings were determined (between approximately 20–95%), awareness was highest in uterine prolapse and lowest in pelvic inflammatory disease. Uterine prolapse is a likely complication of childbirth from weakness in the pelvic floor muscles, and physiotherapy as a conservative management in the form regular pelvic floor exercises can be initiated in the immediate postpartum period [19, 20]. Hence, the finding of high awareness of physiotherapy’s role in postnatal care and uterine prolapse treatment is appreciable. A similar study conducted in Nigeria also reported high awareness levels in postnatal care and uterine prolapse [4]. It is however important to emphasize that high level of awareness of physiotherapy relevance in maternal health is not directly translated to optimal clinical utilization in terms making timely referrals of postnatal mothers for physiotherapy services.

Intriguingly, major variation in level of awareness was determined among the categories of doctors in this study. For instance, consultants demonstrated the highest (85.7%) awareness of the role of physiotherapy in antenatal care and most gynecological conditions compared to the residents (78.8%). The high level of awareness regarding the role of physiotherapy in women’s health demonstrated by the consultants is partly attributed to their extended duration of practice, varied clinical exposures and experience. In Ethiopia, Kutty reported similar findings and attributed the level of awareness to clinical experience and longer period of exposure to cases requiring physiotherapy [24]. Previous studies have determined that doctors’ characteristics such as years in practice greatly influence their level of awareness [4, 24]. In our study, the duration of clinical practice (≥ 10years) significantly increased the odds of doctors’ awareness regarding the importance of physiotherapy in childbirth (odd ratio = 3.5) only but not in other clinical areas. However, the statistical significance disappeared after adjusting for the relevant confounders. Therefore, further research with a larger sample size is recommended to evaluate this association.

Regarding attitude towards physiotherapy, majority of the obstetricians had a positive attitudes towards physiotherapy although areas of negative attitudes were also recorded. For instance, only 19.7% of obstetricians strongly agreed to physiotherapists involvement during labour. This finding partly accounts for the low level of awareness of the relevance of physiotherapy during parturition as compared to the other categories in maternal health care. The finding of low awareness on the part of obstetricians concering the need for active participation of physiotherapists in the management of labour and delivery is intriguing. Generally, continuous support during childbirth is strongly recommended for women because of its association with improved birth outcomes [25] and physiotherapists are recommended as major contributors. Likewise, it is imperative that physiotherapy services are made freely available to women in labour to reinforce the education received during antenatal period and supplement the non-pharmacological pain management in labour.

Furthermore, over 30% of the obstetricians/gynecologists disagreed that physiotherapists had been effective in their inter-personal relationship with other health professionals. This finding may be due to complaints raised about physiotherapists’ infrequent availability at ward rounds. The opinions of the doctors on physiotherapy practice clearly reveals their inherently low impression about the professional scope of physiotherapy. There is the need for physiotherapists to create more awareness regarding the scope of physiotherapy in the multidisciplinary team comprising obstetricians/gynecologists, nurses, and midwives. Inter-professional education may improve collaboration among members of the multidisciplinary team and facilitate effective and efficient team work resulting in improved quality of care [26]. More recently, Goyekar and Shah recommended that regular professional communication and improved interaction between obstetricians/gynaecologists and physiotherapists may improve the utilization of physiotherapy in women’s health [8].

In a similar study in Nigeria, Odunaiya et al concluded that having high awareness does not necessarily translate into having positive attitude [4]. In this study, factors influencing utilization of physiotherapy services were explored and most of the spectrum supported the high awareness and utilization physiotherapy in women’ health. This is in line with the study by Sangal et al who reported that knowledge about a service is a very vital factor in determining its utilization [27]. It is important to constantly showcase the availability of the various physiotherapy services in the hospital to the various medical specialties to enhance optimal utilization and early referral for physiotherapy. This will obviously encourage active participation of physiotherapists in all aspect of women’s health where the involvement of physiotherapy services is vital.

Most of the obstetricians/gynecologists had previously worked with physiotherapists in the management of obstetric and gynecological patients. This previous working experience accounts partly for their high attitude towards the involvement of physiotherapy in the management of specific obstetric and gynecologic cases. Nevertheless, nearly 50% of the doctors reported that there are limited numbers of physiotherapists to cover the obstetrics and gynecology wards which in turn affects the overall utilization of physiotherapy. The reason for this may be due to low recruitment rate of physiotherapists into government hospitals and lack of adequate number of facilities for training physiotherapists. To buttress this point, only 6.6% of the doctors had ever attended ward rounds with physiotherapists. This suggests that there is suboptimal co-ordination and lack of functioning multi-disciplinary approach to clinical management, resulting in suboptimal quality of care for women’s conditions which require physiotherapy services. There is the need to urgently create more awareness about the critical importance of physiotherapy in women’s health.

### Clinical and research implications

**Fig. 2:**
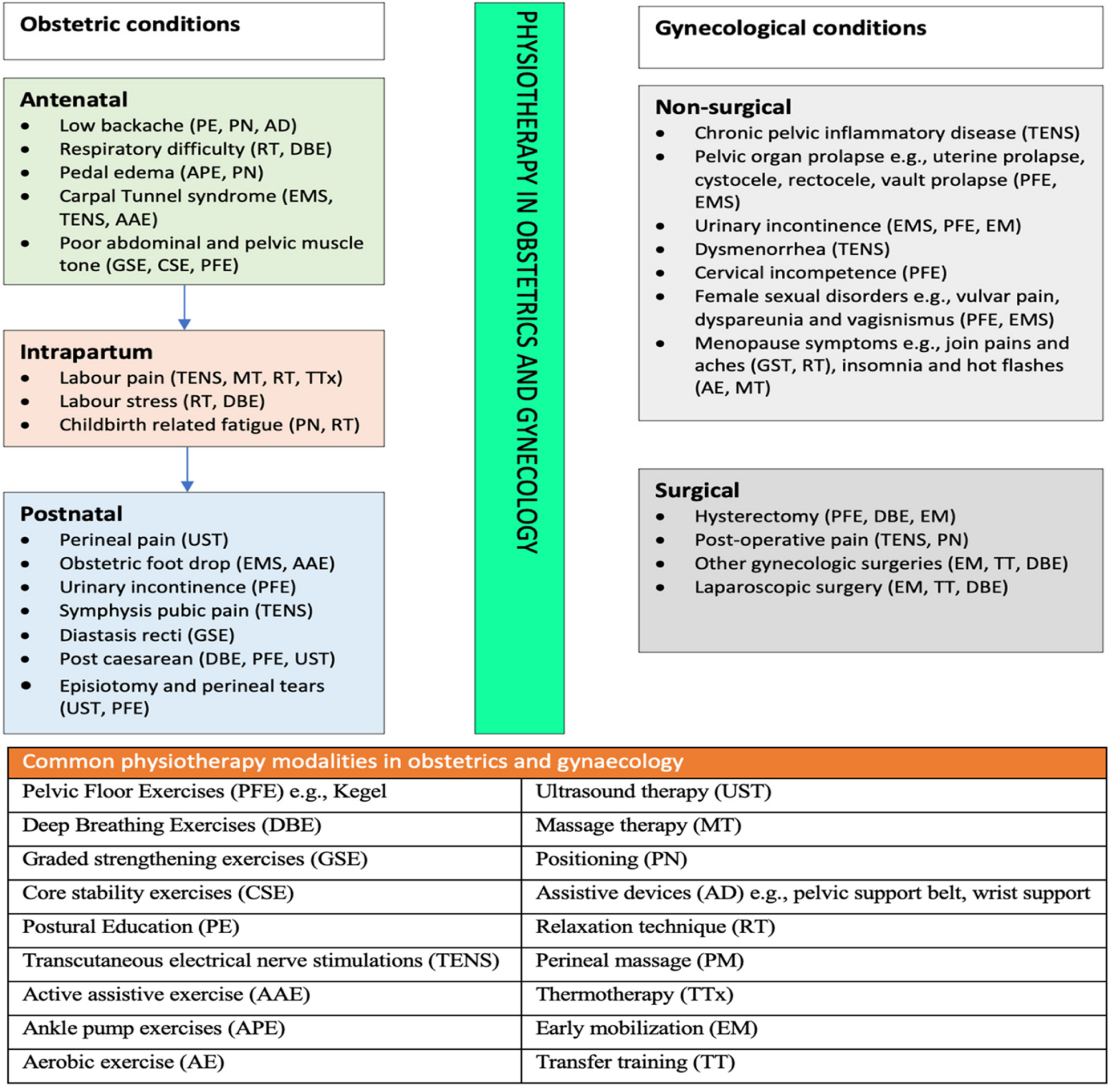
Common conditions in obstetrics and gynecology indicated for physiotherapy

Our study indicates that physiotherapy remains a vital adjunct in the management of common conditions in obstetrics (antenatal, intrapartum and postnatal including post caesarean section) in accordance with other studies [6, 28] and gynecology (surgical and non-surgical) [29]. Figure 2 highlights the common obstetric and gynecological conditions which require physiotherapy services and the available physiotherapeutic modalities [6, 28, 29]. There is urgent need to actively integrate physiotherapy services into women’s health care with regular monitoring and evaluation of its impact on the quality of care women experiences. Proactive integration of coordinated inter-professional education through advocacy and workshops involving the obstetric/gynecological multidisciplinary teams is vital in optimizing utilization of physiotherapy in women’s  health care. The need for recruitment of more physiotherapists in government hospitals and to provide continuous professional training opportunities is well acknowledged to ensure improved quality of care in women health. A recent qualitative research showed that several factors influence women’s adherence to pelvic floor exercises and these include effective physiotherapy programs, their personal experiences, awareness or beliefs and professional feedback  [30]. This evidence supports the immense role of physiotherapists to women’s health and the urgent  need for its optimal integration to improve  the quality of care for women requiring such adjunctive care. Further research (including qualitative design) of high methodological quality relating to the role of physiotherapy in the practice of obstetrics and gynecology is strongly recommended. Special areas of research include assessing implementation challenges associated with regular utilization of physiotherapy services in women’s health. In additions, research involving the opinions of relevant stakeholders including women and other health professionals (nurses and midwives) is recommended to facilitate efficiency of physiotherapy practice in women’s health.

The strength of the study relates the fact that it is the first study conducted to assess, obstetricians/gynecologists’ awareness, attitudes and utilization of physiotherapy in women’s health in Ghana. The findings will serve as a baseline information for further studies on physiotherapy in women’s health. The small numbers of study participants involved in the study constitutes a major limitation and might have influenced the findings determined. The study employed mostly close-ended questions which narrowed the doctors opinions and concerns about the physiotherapy profession. The doctors could not describe their own experiences concerning the role of physiotherapy in women’s health as qualitative research design would have offered and this constitute a significant limitation. Also, non-inclusion of other health professionals such as nurses and midwives providing maternity care services is considered a limitation of the study as the responses by only the doctors might be skewed.

## Conclusion

Most of the obstetricians and gynecologists showed high awareness levels towards physiotherapy services in women’s health. Overall, the consultants showed high awareness levels compared to the resident doctors in antenatal and gynecological care whiles senior residents had more awareness in intrapartum and postnatal care, although these were not statistically significant. Junior residents generally showed the lowest awareness levels compared to consultants and senior residents. Clinical practice duration ≥ 10 years was not significantly associated with increased the odds of doctors’ awareness concerning the relevance of physiotherapy in childbirth and other clinical areas. There was mixed findings concerning the doctors’ attitudes toward physiotherapy in women’s health. Factors influencing the utilization of physiotherapy services include non-availability of enough physiotherapists and failure of physiotherapists to attend ward rounds to enhance education on the scope of physiotherapy practice in women’s health.

## Data Availability

The data collected for this study can be obtained from the first author based on a reasonable request from the corresponding author.
